# Glycolysis in the African Trypanosome: Targeting Enzymes and Their Subcellular Compartments for Therapeutic Development

**DOI:** 10.4061/2011/123702

**Published:** 2011-04-11

**Authors:** April F. Coley, Heidi C. Dodson, Meredith T. Morris, James C. Morris

**Affiliations:** Department of Genetics and Biochemistry, Clemson University, Clemson, SC 29634, USA

## Abstract

Subspecies of the African trypanosome, *Trypanosoma brucei*, which cause human African trypanosomiasis, are transmitted by the tsetse fly, with transmission-essential lifecycle stages occurring in both the insect vector and human host. During infection of the human host, the parasite is limited to using glycolysis of host sugar for ATP production. This dependence on glucose breakdown presents a series of targets for potential therapeutic development, many of which have been explored and validated as therapeutic targets experimentally. These include enzymes directly involved in glucose metabolism (e.g., the trypanosome hexokinases), as well as cellular components required for development and maintenance of the essential subcellular compartments that house the major part of the pathway, the glycosomes.

## 1. Introduction

African sleeping sickness is considered a “neglected tropical disease” yet continues to be a major public health risk to sub-Saharan Africa. A survey from 2005 analyzed by the World Health Organization indicated that African sleeping sickness was still prevalent, with an estimated 50,000 to 70,000 cases occurring (http://www.who.int/mediacentre/factsheets/fs259/en/). A survey from 2009 suggests that the number of cases is falling, but the current level of disease management requires stable social conditions for accurate surveillance and control measures to be effective. Further, the lack of safe and efficacious treatments emphasizes the need for research on new therapies. The current drugs used to treat the disease are often toxic, and their administration typically requires skilled medical care. Additionally, some of the compounds fail to function against certain subspecies, and resistance is a growing concern.

The parasite is transmitted by the bite of the blood-feeding tsetse fly and initially causes fever, headache, and joint pain in humans. Winterbottom's sign, a swelling of the lymph nodes characteristic of early trypanosome infection, has long been recognized in association with African trypanosome infection—slave traders in the 1800s would relocate their operations within Africa upon its appearance in populations destined for slavery [1]. 

As the disease progresses, parasites enter the brain, and neurological symptoms, such as confusion, disturbed sleep patterns, extreme lethargy (hence, “sleeping sickness”), and coma occur. Left untreated, the disease is invariably fatal. Annual death numbers as a result of African sleeping sickness are difficult to determine, as limited monitoring in rural Africa likely leads to underestimated infection rates. 

Human health is also impacted indirectly by the parasite, as animals used for food are also subject to infection. An infected animal experiences fever, listlessness, emaciation, and paralysis, leading the animal to be unfit for use, hence the term “nagana” which is a Zulu word that means “powerless/useless” [1]. It is estimated that 3 million cattle die each year from this disease (Food and Agriculture Organization of the United Nations, http://www.fao.org/). The prevalence of nagana in animals renders much of the African continent inhospitable for livestock production, with an area equal to the continental US unsuitable for beef or dairy production. 

Essential lifecycle stages occur in both the vector and mammalian host. In the fly midgut, parasites taken up during a blood meal differentiate into procyclic form (PF) parasites. These parasites escape the peritrophic membrane and invade the surrounding tissues. Coincident with this behavior, the parasites differentiate into an epimastigote form, which then infects the salivary glands. Once in the salivary glands, parasites develop into nonproliferative metacyclic trypanosomes that are competent for establishing infection in the mammalian host. Delivery of the trypanosome to the mammal occurs when the fly feeds again. Bloodstream form (BSF) parasites develop and grow rapidly in the host blood, with a portion of the population developing into short stumpy parasites that, when taken up by a feeding fly, continue the lifecycle.

Lifecycle stages take advantage of distinct niches to fulfill their metabolic needs. PF parasites utilize the abundant amino acids in their surroundings to generate ATP through mitochondrial-based pathways. While glycolysis is important to the PF parasites, these parasites can thrive in the absence of glucose if adapted to low-glucose conditions, indicating that other metabolic pathways can compensate for the loss of glycolysis [2, 3]. 

In BSF parasites, glycolysis of host glucose provides the sole source of carbon for ATP production. This dependence on glycolysis for ATP coincides with reduced mitochondrial function, limiting the metabolic options available to the parasite and presenting a series of targets for potential therapeutic development. These include enzymes that participate directly in glycolysis, proteins responsible for enzyme import into glycosomes, and cellular components involved in the regulation of glycosome number and differentiation. Here, we discuss targeting enzymes of glycolysis, with a particular focus on the first enzyme in the pathway, *T. brucei *hexokinase 1 (TbHK1). Additionally, compartmentalization of the pathway is critical to the success of the parasite, so we will consider strategies aimed at disruption of mechanisms the parasite uses during the maturation and development of glycosomes.

## 2. Glycolysis in the BSF African Trypanosome

**Figure 1:**
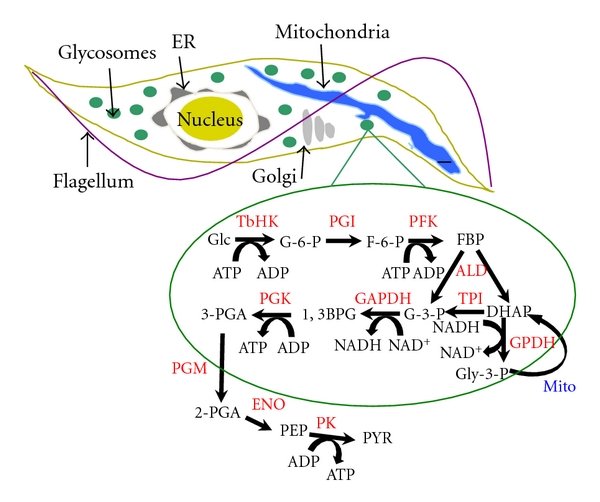
Glycolysis and glycosomes in the bloodstream form African trypanosome. Abbreviations: ALD: aldolase; DHAP: dihydroxyacetone phosphate; 1,3BPGA: 1,3-bisphosphoglycerate; ENO: enolase; F-6-P: fructose-6-phosphate; FBP: fructose 1,6-bisphosphate; G-3-P: glyceraldehyde 3-phosphate; G-6-P: glucose-6-phosphate; GAPDH: glyceraldehyde-3-phosphate dehydrogenase; Glc: glucose; Gly-3-p: glycerol-3-phosphate; GPDH: glycerol 3-phosphate dehydrogenase; Mito: mitochondrial enzymes; PEP: phosphoenolpyruvate; 2-PGA: 2-phosphoglycerate; 3-PGA: 3-phosphoglycerate; PGI: glucose-6-phosphate isomerase; PGM: phosphoglycerate mutase; PFK: phosphofructokinase; PGK: phosphoglycerate kinase; PK: pyruvate kinase; PYR: pyruvate; TbHK: *T. brucei *hexokinase 1 and/or 2; TPI: triose-phosphate isomerase.

Metabolism of host glucose through glycolysis is essential to the success of a BSF parasite mammalian infection, as the pathway is the sole source of ATP production in the mammalian infection lifecycle stage. The pathway is organized into subcellular compartments related to peroxisomes named glycosomes. First characterized in 1977 by Opperdoes and Borst, the single-membrane compartment houses the first seven enzymes of glycolysis [4]. Under aerobic conditions, these enzymes convert glucose to 3-phosphoglycerate, which is then further metabolized to pyruvate with the concomitant production of ATP by pyruvate kinase in the cytosol (Figure 1). The pyruvate is then secreted from the cell. 

One key to the presence of compartmentalized glycolysis is related to regulation of energy metabolism. ATP and reducing equivalent depletion and production within the glycosome are balanced. ATP is consumed by the activity of the TbHKs and phosphofructokinase (PFK), while it is regenerated by the activity of the glycosomal phosphoglycerate kinase (gPGK). Additionally, NADH produced by glyceraldehyde-3-phosphate dehydrogenase is balanced by NADH oxidation when glycerol 3-phosphate dehydrogenase (GPDH) metabolizes dihydroxyacetone phosphate (DHAP) to glycerol 3-phosphate (Gly-3-p). The resulting Gly-3-p is shuttled from the glycosome to the mitochondria where electrons are ultimately transferred to water through the activity of a glycerol 3-phosphate oxidase complex (consisting of a mitochondrial glycerol 3-phosphate dehydrogenase, ubiquinone, and trypanosomal alternative oxidase). The shuttle returns DHAP to the glycosome, allowing maintenance of the glycosomal redox balance. 

The compartmentalization of a majority of the glycolytic pathway segregates important steps in the path to ATP synthesis and creates what could be considered additional obstacles to efficient energy metabolism. Why does the parasite do this? Bakker and colleagues, through a combination of computational and wet-bench experiments, have found that compartmentalization of glycolytic enzymes that lack allosteric regulation prevents the unchecked consumption of ATP in a “turbo-explosion” of glycolysis [5]. That is, because feedback inhibition does not limit TbHK and PFK activity, these enzymes would generate products (hexose phosphates) at levels beyond the capacity of the downstream enzymes if unchecked by compartmentalization.

## 3. TbHKs as Targets for Therapeutic Development

In the African trypanosome, TbHK, an activity composed of an unknown ratio of two proteins (TbHK1 and TbHK2), mediates the first step in glycolysis. Because the enzymes have the hallmarks of good targets for therapeutic development, considerable effort has been directed toward the development of TbHK inhibitors as potential antiparasitic compounds. First, both TbHK1 and TbHK2 are essential to the BSF parasite, as demonstrated by targeted gene silencing using RNAi constructs specific to the unique 3′ UTRs of the genes [6, 7]. In both cases, cell toxicity was observed after 3–5 days of RNAi exposure. Second, chemical inhibitors of TbHK1 are toxic to the parasite [7–9]. Third, TbHK1 is likely different enough from host enzymes, sharing only 30–33% sequence identity with mammalian HKs, to suggest that it can be specifically targeted. Last, TbHK1 has unusual properties, including oligomerization into hexamers [10] and is inhibited by compounds distinct from those which inhibit the mammalian enzymes, including fatty acids, to suggest that specific inhibition is possible. 

### 3.1. TbHK1 Inhibitors: Approaches for Discovery

Willson et al. developed structural-based inhibitors of TbHK that were antitrypanosomal through modeling of TbHK1 to known HK structures [9]. These glucosamine derivatives were tested and found to be competitive with respect to glucose, with *K*_*i*_ values similar to the *K*_*M*_ value for glucose [9]. However, the compounds were not particularly toxic to BSF parasites (with LD_50_s in the range of 5–10 mM, and an LD_100_ for the best inhibitor of 3.6 mM), possibly because the compounds entered the cell by passive diffusion instead of import against a concentration gradient. Alternatively, the compounds may have been imported by facilitated transport through the glucose transporter, again failing to accumulate to sufficient concentrations for toxicity.

TbHK1 inhibitors have also been identified in surveys of chemicals that inhibit HKs from other systems. The activity of molecules identified by this approach is likely the result of conserved structural features of mammalian and trypanosome HKs. For example, the anticancer drug lonidamine (LND, 1-(2,4-dichlorobenzyl)-1,H-indazol-3-carboxylic acid), which inhibits human HK and has been subject to clinical trials in humans also inhibits both recombinant TbHK1 and TbHKs from parasite lysate and is toxic to the parasite [7, 11–13]. Additionally, quercetin (QCN, 3,5,7,3′,4′ pentahydroxyflavone), which inhibits a number of mammalian enzymes including HKs, is toxic to *T. brucei* and inhibits recombinant TbHK1 through binding near the TbHK1 active site [14–16].

Lack of sensitivity of the trypanosome enzymes to other known HK inhibitors, including glucose-6-phosphate, 5-thio-D-glucose, and 3-methoxyglucose, suggests that the TbHKs are sufficiently unique for therapy development [7]. A group of bisphosphonates that are potent inhibitors of *T. cruzi* HK did not inhibit rTbHK1, emphasizing the unique nature of the TbHKs [17, 18]. Notably, rTbHK2, when oligomerized *in vitro *with a catalytically inactive rTbHK1 variant, is active, and the activity is sensitive to PPi inhibition and, to a lesser extent, the bisphosphonate risedronate [10].

The potential arsenal of leads has recently been expanded using two screens to identify specific inhibitors of recombinant TbHK1. The first screen, of a library of pharmacologically active compounds (LOPAC), yielded 18 primary hits (>40% inhibition at 10 *μ*M) from 1280 compounds, including myricetin, a bioflavonoid that is structurally very similar to QCN [19]. In addition to the identification of new lead compounds, the LOPAC screen served to validate the conditions required for automated high-throughput screening (HTS) of a 220,233 compound library.

The HTS campaign initially yielded 239 compounds as primary actives (>50% TbHK1 inhibition at 10 *μ*M), which were then cherry-picked and confirmed as TbHK1 inhibitors. Thirteen compounds with IC_50_ values <50 *μ*M were purchased from commercial sources and ten confirmed with IC_50_ values <50 *μ*M. Of these ten, six clustered into a structurally related group (isobenzothiazolinones), and four were singletons. These compounds had IC_50_s that ranged from 0.05–41.7 *μ*M, and some of the TbHK1 inhibitors were toxic to BSF *T. brucei*, with EC_50_ values of 0.03–2.9 *μ*M while not exhibiting toxicity towards mammalian cells [19]. 

In summary, TbHK1 has served as a viable target for therapeutic lead development, with the exciting possibility of the development of potent target-specific inhibitors indicated by recent HTS results. These findings are in agreement with studies that considered the consequences of reduced glycolytic flux through inhibition of the TbHKs on trypanosome growth. Initial *in silico* studies predicted that the TbHKs (and several other glycolytic enzymes) were present in excess, suggesting that significant inhibition would be required to yield a detrimental impact on glycolytic flux and, therefore, parasite health [20]. However, refinement of the model combined with additional experimental assessment revealed that TbHK and PYK were less abundant than initially thought, and that partial inhibition of the enzymes could sufficiently reduce flux to toxic levels in the parasite [6].

### 3.2. Other Glycolytic Enzymes as Targets

Could other enzymes in glycolysis be targeted for therapeutic development? The other *T. brucei* HK, TbHK2, is 98% identical to TbHK1, so it is likely that compounds that inhibit TbHK1 would also impact TbHK2, though the lack of *in vitro *HK activity has limited studies into this possibility [21]. Downstream, other enzymes have limited identity to human proteins, and several have been validated genetically or chemically as drug targets (Table 1). For a review of the potential of other glycolysis enzymes as therapeutic targets, please see [22, 23].

Mechanisms of regulation of glycolytic enzyme expression may yield interesting targets. In the case of the TbHKs, it has been established that (1) either reduced or excessive expression of TbHK is toxic to the parasite [6, 7], and (2) the environment in which the parasite is grown influences TbHK expression [10]; however, the molecular mechanisms that allow precise yet regulable expression remain unresolved.

## 4. Glycosomal Glycolytic Enzyme Import: Targeting the Machinery

Glycosomal resident proteins are encoded by nuclear DNA, translated on cytosolic polyribosomes and targeted to glycosomes as a result of bearing a glycosomal targeting sequence. Proper glycosomal targeting is essential to the parasite because otherwise glucose is toxic to the parasite. RNAi of PEX14, a peroxin required for glycosome protein import, led to accumulation of glycosomal resident proteins in the cytosolic fraction. This condition was tolerated by PF parasites unless they were cultured in the presence of glucose. If grown with glucose, the PEX14-deficient cells accumulated glucose-6-phosphate, fructose-6-phosphate, and fructose-1,6-bisphosphate and died [37–39]. Notably, depletion of TbHK in the PEX14-deficient parasites through simultaneous RNAi of the TbHKs and PEX14 yielded cells that were no longer sensitive to glucose, suggesting that the compartmentalization of glycolysis (or the TbHKs) is essential [38]. Additionally, expression of a targeting-deficient HK in *L. donovani *was lethal to parasites in the presence of glucose [40]. While the observed parasite death may have resulted from unchecked ATP consumption, the observation that TbHK1 is regulated by a number of other mechanisms suggests that this may not be the sole explanation for the observed glucose toxicity [10, 21].

Three types of targeting sequences are known to mediate targeting to glycosomes. These sequences, that share similarity with peroxisomal targeting sequences (PTS), include the PTS1, PTS2, and an I-PTS (internal-PTS). Enzymes that participate in glycolysis have PTS1, PTS2, and I-PTS targeting sequences (Table 1). 

The PTS1 and PTS2 targeting sequences have been well characterized while less is known about the I-PTS. PTS1 is a C-terminal three amino acid sequence originally identified in firefly luciferase [41]. PTS1-bearing proteins are localized to peroxisomes (and glycosomes) through an interaction with the peroxin protein PEX5, with PTS1 recognition occurring through signal sequence interaction with seven predicted tetratricopeptide repeats in the PEX5 [42]. 

The PTS2 was first identified when mutations in the N-terminus of the rat peroxisomal 3-ketoacyl-CoA thiolase precursor led to mislocalization of the protein [43]. Mutagenesis studies revealed that the N-terminus of *Saccharomyces cerevisiae* thiolase (which is identical at 6 of 11 amino acids with the rat thiolase N-terminus is necessary and sufficient for protein targeting to the peroxisomes [44]. Contrary to PTS1 and PTS2 signals, the I-PTS sequences lack obvious similarity, sharing only that they are internally located in a polypeptide [45].

### 4.1. PEX7 and PEX5: Central Participants in Glycosome Targeting

Protein import into the glycosome requires interaction with multiple proteins, including those identified and characterized for peroxisomal import. For example, *S. cerevisiae* PEX7 (originally named PAS7 or PEB1) is involved in transport of PTS2-bearing proteins to the peroxisome [46, 47]. The yeast PEX7 does not require a peroxisomal membrane for binding to the thiolase but binds thiolase in a PTS2-dependent manner. Further, yeast PEX7 does not need a free N-terminus near the PTS2 for binding to occur, and binds thiolase that has already been folded, suggesting that the protein interacts with thiolase in the cytoplasm and acts as a shuttle between the cytoplasm and peroxisome [48].

PEX7 homologs have been identified in three trypanosomatid species, *T. brucei*, *L. major*, and *T. cruzi*. These PEX7 sequences are 65–76% identical to one another and 32–36% identical to the human and *S. cerevisiae* proteins. The trypanosomatid PEX7s contain a C-terminal proline-rich ~40 amino acid extension while the equivalent human and yeast structures have a shorter (5 and 10 residues, resp.) extension that lacks the proline enrichment. 

**Figure 2:**
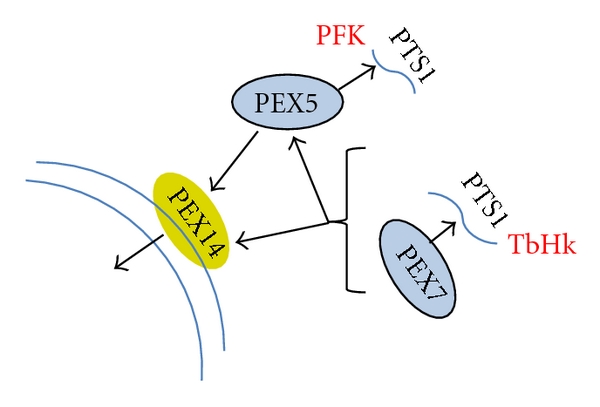
PTS binding proteins participate in delivery of glycolytic enzymes to the glycosome. Fully folded PTS2 harboring proteins expressed in the cytoplasm, like the TbHKs, are targeted to the glycosome through the binding of PEX7 to the PTS2. This complex may or may not interact with PEX5 prior to delivery to PEX14 for transfer to the glycosome matrix. PFK, which harbors an internal PTS1 targeting sequence, is targeted by PEX5.

In mammals, PEX7 bound to PTS2 proteins interacts with another peroxin, PEX5, for import into peroxisomes [49, 50]. In 2008, recombinant *L. major* PEX7 was expressed and purified, and this protein was shown to bind to PTS2 sequences [51]. LmPEX7 also binds to a polypeptide derived from *L. donovani* PEX5 (LdPEX5). Other trypanosomatids, including *T. brucei *and *T. cruzi*, also harbor a PEX5 homolog that contains a putative PEX7 binding box located in the N-terminal half of the protein [52]. These findings suggest that the trypanosomatid PEX7 proteins, like the mammalian PEX7 proteins, function through an interaction with PEX5 protein (Figure 2), though RNAi of PEX5 did not alter localization of some PTS2 proteins in *T. brucei, *indicating this relationship may not be an absolute requirement for all PTS2 protein import. 

*T. brucei* PEX5 (TbPEX5) is also involved in the import of PTS1-containing proteins into the glycosome. The PTS1 binding domain of TbPEX5 has been characterized and consists of tetratricopeptide repeats, which typically form super helices that allow protein:protein interactions on both the inner and outer faces [53]. This could allow TbPEX5 to interact simultaneously with multiple proteins [54]. 

In summary, glycosomal resident proteins are compartmentalized as a result of interactions with peroxins in the cytoplasm. PEX7 binds PTS2-bearing proteins, followed by (in some cases) interaction with PEX5, which may also be loaded with PTS1 harboring proteins. This complex is then delivered to the glycosomal membrane where it docks with a glycosomal membrane protein, PEX14, which participates in import of matrix proteins [38]. The mechanisms of import of PTS1 and PTS2 proteins are slightly different, with PTS1-targeted proteins translocated into the glycosome coincident with the release of their PEX5 binding partner back into the cytoplasm. The PEX7:PTS2 protein complex is translocated *en block* into the glycosome where the PTS2 protein partner is released followed by transport of the PEX7 protein out of the glycosome [51]. 

Glycosomal resident matrix proteins are expressed from cytosolic polyribosomes as fully folded polypeptides [55]. This creates a potentially dangerous situation for the cell, as inappropriate cytosolic expression of glycolytic enzymes may be toxic to the parasite [40]. While the mechanisms that maintain enzymes in an inactive state in the cytosol are not known, it is tempting to speculate that interaction with peroxisomal targeting proteins may participate in preventing cytosolic activity. With that in mind, targeted disruption of this relationship, through small molecules that interfere with the protein:protein interactions, for example, could ablate regulation and prevent appropriate subcellular localization—with destructive consequences to the parasite.

## 5. Glycosome Replication and Development as Additional Targets

*T. brucei* must maintain glycosome number and integrity to maintain homeostasis under normal conditions and remodel glycosomal contents during differentiation and in response to changes in environmental conditions. Components that regulate the dynamics of these essential organelles are potential drug targets.

**Figure 3:**
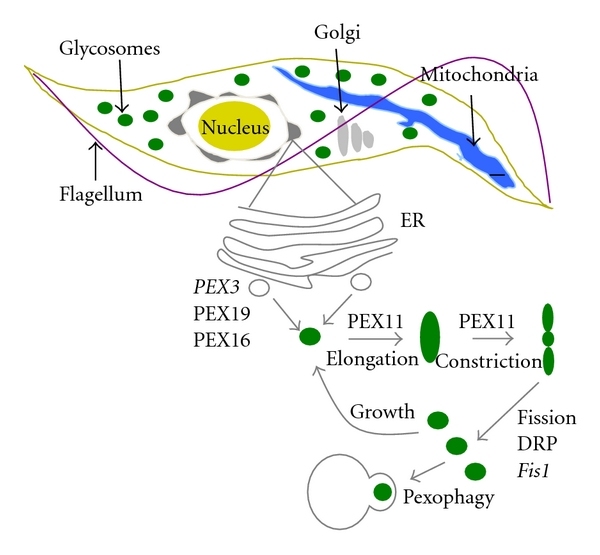
Proposed overview of glycosome biogenesis and remodeling. Proteins without obvious *T. brucei* homologs are indicated in italics.

Glycosome biogenesis involves organelle formation, import of proteins from the cytoplasm (see above), proliferation, and remodeling (Figure 3). Rapid advances in cell biology have facilitated the study of peroxisome dynamics in yeast and other model systems, while less is known about these processes in *T. brucei*. Some peroxisome biogenesis protein gene homologs are readily evident in the *T. brucei* annotated genome while others either lack sufficient conservation for identification or are absent. In some cases, homology searches may be hampered because the parasites have streamlined glycosome biogenesis and do not carry out all of the processes observed in the regulation of peroxisomes in other systems. 

### 5.1. De Novo Growth of Peroxisomes

Peroxisomes can proliferate through *de novo* budding from the ER and/or by growth and fission of existing organelles. The extent to which process predominates is unclear but appears to vary from organism to organism and is influenced within a given species by growth conditions. 

In *S. cerevisiae*, *de novo *peroxisome formation involves the integral membrane protein PEX3, which localizes to the endoplasmic reticulum, forming distinct foci that interact with the peroxisomal membrane protein PEX19. The PEX3/PEX19 vesicles bud from the ER and mature into functional peroxisomes [56]. In support of the ER to peroxisome maturation model, sixteen different peroxisomal membrane proteins were found to localize to the ER in *S. cerevisiae* via traditional ER translocation machinery [57]. In mammalian cells, an additional protein PEX16 (not present in yeast) is involved in formation of peroxisomes from ER in the absence of pre-existing organelles [58, 59].

It is unknown if *de novo *glycosome formation occurs in *T. brucei*. To date, no homologs for PEX3 have been identified in* T. brucei,* although it has been proposed that, through gene displacement, the parasite has developed an alternative replacement activity, as the function of this protein in glycosome biogenesis is likely essential [60]*.*

A PEX19 homolog, on the other hand, has been identified in *T. brucei*. The protein, TbPEX19, exhibits low sequence identity (18–22%) to PEX19 from other organisms and was identified only when relaxed BLAST searches were employed [61]. TbPEX19 is essential in *T. brucei *and is involved in glycosomal protein import with specificity that is similar, though not identical, to that observed for yeast and human PEX19 [62]. Its role in *de novo* formation of glycosomes has not been assessed.

### 5.2. Growth and Fission of Existing Organelles: The Role of PEX11 in Early Division

In addition to ER-dependent formation of peroxisomes, peroxisome proliferation can also occur through the growth and division of existing organelles. The early process of elongation and constriction of peroxisomes involves PEX11 while the later process of fission involves a set of dynamin-related proteins (DRPs). 

PEX11-family proteins, the first proteins to be implicated in peroxisome division, are present in all eukaryotic cells [63, 64]. All PEX11 homologs are ~25 kDa, with isoelectric points greater than 9 and significant sequence similarities at their N- and C-termini. The *S. cerevisiae *PEX11 family includes PEX11, PEX25, and PEX27 [65]*. A. thaliana* contains five PEX11 isoforms (PEXa-e), while mammals have three (PEX11 *α*, *β*, *γ*) [66–68]. *T. brucei* PEX11 family proteins include TbPEX11 as well as two PEX11-like genes, TbGIM5A and TbGIM5B [69, 70].

In *T. brucei*, TbPEX11, TbGIM5A, and TbGIM5B are all associated with the glycosomal membrane via two transmembrane (TM) domains leaving the N- and C-termini exposed to the cytoplasm [69, 70]. TbGIM5A and TbGIM5B are 97% identical with the amino acid differences found within the sequence that links the two TM domains [70]. Like TbPEX11, antiserum that recognizes TbGIM5A and TbGIM5B cross-reacts with proteins that localize to glycosomes, and depletion of this protein results in altered glycosome morphology.

PEX11 proteins undergo a number of posttranslational changes including dimerization and phosphorylation. In *S. cerevisiae* PEX11, homodimers are enriched in mature peroxisomes, and inhibition of this dimerization results in the overproliferation of peroxisomes [71]. TbPEX11, TbGIM5A, and TbGIM5B also form homodimers while TbGIM5A and TbGIM5B form heterodimers with each other but do not interact with PEX11 [70]. The functional significance of this interaction in *T. brucei* is unknown.

*S. cerevisiae *PEX11 is reversibly phosphorylated at Ser165 and Ser167 [72]. Expressing constitutively dephosphorylated PEX11 results in cells containing fewer, larger peroxisomes while constitutively phosphorylated PEX11 results in enhanced peroxisome proliferation. There is no experimental evidence that TbPEX11 is phosphorylated *in vivo*. Sequence analysis using NetPhos 2.0 (http://www.cbs.dtu.dk/) predicts five potential Ser phosphorylation sites (at residues 42, 50, 154, 159, and 194) and three potential Thr phosphorylation sites (residues 158, 196, and 197). 

In fungi, plants, mammals, and *T. brucei*, PEX11 reduction results in cells that contain fewer, larger peroxisomes as compared to wild-type cells [63, 65, 66, 68, 69]. Likewise, increased expression results in the production of smaller peroxisomes in greater abundance than found in normal cells [64–66, 69, 73].

One kinase involved in the phosphorylation of PEX11 is Pho85, a cyclin-dependent kinase. *S. cerevisiae* strains lacking Pho85 had few, larger peroxisomes as compared to parental yeast while cells overexpressing Pho85 had hyperphosphorylated PEX11 [74]. The Pho85 overexpressing yeast also demonstrated increased rates of peroxisome proliferation in comparison with wild-type cells, suggesting that Pho85 plays a role in regulation of peroxisome proliferation [72].

### 5.3. Growth and Fission of Existing Organelles: The Role of DRPs in Late Division

Peroxisome fission is regulated by a number of dynamin-related proteins (DRPs), which are large GTPases involved in membrane fission and fusion. The peroxisome fission machinery was first identified through studies of mitochondrial fission. In yeast, there are two DRPs, Vps1 and Dnm1, involved in peroxisome fission (for reviews, see [75, 76]). The extent to which each functions is dependent on the organism as well as growth conditions. In *S. cerevisiae*, the Vsp1 dependent system prevails under conditions in which peroxisome proliferation is repressed while the Dnm1 pathway predominates when peroxisome proliferation is induced [77]. *T. brucei* harbors a single DRP, TbDLP, although its role in peroxisome division has not been investigated [78, 79].

DRPs are targeted to the peroxisome membrane through a series of protein-protein interactions. In yeast, Dnm1 is targeted to the peroxisome membrane via interaction with Fis1, a tail anchored protein that has been found to localize to both the mitochondria and peroxisomes [77, 80, 81]. In yeast, Dnm1 is bound to Fis1 through the adaptor proteins Mdv1/Caf4 [80, 82]. In mammals, this adaptor function is likely performed by another set of proteins as no Mdv1/Caf4 homologs have been identified. Instead, mammals target Fis1p to the peroxisome via PEX11*β* [83]. Vps1 functions independently of Mdv1/Caf4 and Fis1, being targeted to peroxisomal membranes via PEX19 [84]. 

Peptide antibodies generated against residues 12–25 of TbDLP labeled both mitochondria and glycosomes, though the glycosomal localization may be an artifact of the highly distributed mitochondria [78]. Silencing the TbDLP gene in PF parasites reduced growth rates and resulted in mitochondrial abnormalities with little effect on other organelle morphologies [78]. In another study, silencing TbDLP again resulted in abnormal mitochondrial morphology with no obvious effect on glycosome morphology [79]. The lack of obvious glycosome defects may be a result of the essential nature of the organelle under these conditions. In standard procyclic media containing glucose, glycosome defects are lethal and would not be available for analysis.

### 5.4. Remodeling of Glycosome Protein Composition: Peroxisome Specific Autophagy

Peroxisomes can be selectively degraded through a conserved mechanism of selective autophagy termed pexophagy. Microscopic observation of *T. brucei* undergoing differentiation of BSF to PF parasites revealed a population of glycosomes that associated with the lysosome. This association is concomitant with changes in the expression of glycosome proteins and suggests that this turnover of glycosomes may occur through a process analogous to pexophagy [85]. Recent bioinformatic analysis has identified trypanosome homologs for about one-half of the known autophagy components from yeast. See [60] for a discussion of the proteins involved in autophagy and their trypanosome homologs.

### 5.5. Targeting Glycosome Dynamics with Therapeutics: Challenges and the Future

Our understanding of *T. brucei* glycosome dynamics and biogenesis is limited, particularly when compared to what is known about the regulation of peroxisomes from other systems. This is in part due to the unusual properties of the glycosome—it differs functionally from peroxisomes in a number of ways that are not limited to compartmentalization of glycosomes. These differences yield a compartment that is regulated by means distinct from peroxisomes—many of the key proteins involved in these processes lack homologs in other systems. To overcome this obstacle, one could envision applying the power of forward genetics, a tool that has been deployed in the study of the African trypanosome, to identify cellular mechanism that regulate glycosome dynamics [24]. These genes will include many parasite-specific, essential regulators of glycosome biology—which will add to the list of interesting therapeutic targets.

## 6. Conclusions

Glycolysis and mechanisms required for its compartmentalization remain attractive targets for therapeutic development. Specific inhibitors of parasite glycolytic enzymes have been identified, suggesting that differences, though they may be slight, are sufficient between mammalian and trypanosomal components for development of novel agents. Pathways involved in import of glycolytic enzymes into the glycosomes are being elucidated, and these present interesting targets for development, given the toxicity of mislocalization of these activities. Lastly, resolving mechanisms behind the control of dynamic developmental regulation of glycosomes may yield additional means of disrupting glucose metabolism in the cell, a prospect we look forward to tackling.

## Figures and Tables

**Table 1:** The *T. brucei *glycolytic enzymes as potential drug targets.

Enzyme^a^	PTS type	% identity to human counterpart	Status of therapeutic development^b^
TbHK1	PTS2 [24]	38% to HKDC1	CV [7, 9], GV [7, 9],
		36% to HXK3	
TbHK2	PTS2 [24]		GV [6, 7]
PGI	PTS1 [25]	57% to PGI isoform 2	
PFK	PTS1 [25]	27 % to PFK, platelet isoform	CV [26], GV [6]
ALD	PTS2 [26]	49% to brain (C isozyme)	CV [27], GV [28]
TPI	I-PTS [29]	54% to isoform 1	GV [30]
GPDH	PTS1 [25, 31]	38% to GPDH2	
GAPDH	PTS1 [25]	55% to spermatogenic GAPDH-2	CV [32], GV [28]
PGK			
PGKA	I-PTS [33]	42% to PGK 1	
PGKB	N/A	43% to PGK 1	
PGKC	PTS1 [25, 34]	44% to PGK 1	GV [35], CV [36]
PGM	N/A	24% to CAMTA1	GV [6]
ENO	N/A	63% to ENO2	GV [6]
PK	N/A	50% to PKLR	

^a^For enzyme abbreviations, see Figure 1. CAMTA1: calmodulin binding transcription activator 1; HKDC1: hexokinase domain containing protein 1; HXK3: hexokinase type 3; N/A: not applicable because the protein is cytosolic; PKLR: pyruvate kinase, liver, and RBC.

^b^Status: CV: chemically validated target—inhibitors against the target are toxic to parasites; GV: genetically validated target—genetic manipulation of the enzyme leads to growth defects or cell death.

## References

[1] Steverding D (2008). The history of African trypanosomiasis. *Parasites and Vectors*.

[2] Ter Kuile BH (1997). Adaptation of metabolic enzyme activities of *Trypanosoma brucei* promastigotes to growth rate and carbon regimen. *Journal of Bacteriology*.

[3] Drew ME, Morris JC, Wang Z (2003). The adenosine analog tubercidin inhibits glycolysis in *Trypanosoma brucei* as revealed by an RNA interference library. *Journal of Biological Chemistry*.

[4] Opperdoes FR, Borst P (1977). Localization of non glycolytic enzymes in a microbody like organelle in *Trypanosoma brucei*: the glycosome. *FEBS Letters*.

[5] Bakker BM, Mensonides FIC, Teusink B, Van Hoek P, Michels PAM, Westerhoff HV (2000). Compartmentation protects trypanosomes from the dangerous design of glycolysis. *Proceedings of the National Academy of Sciences of the United States of America*.

[6] Albert MA, Haanstra JR, Hannaert V (2005). Experimental and in silico analyses of glycolytic flux control in bloodstream form *Trypanosoma brucei*. *Journal of Biological Chemistry*.

[7] Chambers JW, Fowler ML, Morris MT, Morris JC (2008). The anti-trypanosomal agent lonidamine inhibits *Trypanosoma brucei* hexokinase 1. *Molecular and Biochemical Parasitology*.

[8] Trinquier M, Perie J, Callens M, Opperdoes F, Willson M (1995). Specific inhibitors for the glycolytic enzymes of *Trypanosoma brucei*. *Bioorganic and Medicinal Chemistry*.

[9] Willson M, Sanejouand YH, Perie J, Hannaert V, Opperdoes F (2002). Sequencing, modeling, and selective inhibition of *Trypanosoma brucei* hexokinase. *Chemistry and Biology*.

[10] Chambers JW, Kearns MT, Morris MT, Morris JC (2008). Assembly of heterohexameric trypanosome hexokinases reveals that hexokinase 2 is a regulable enzyme. *Journal of Biological Chemistry*.

[11] Paggi MG, Fanciulli M, Perrotti N (1988). The role of mitochondrial hexokinase in neoplastic phenotype and its sensitivity to lonidamine. *Annals of the New York Academy of Sciences*.

[12] Floridi A, D’Atri S, Menichini R (1983). The effect of the association of gossypol and lonidamine on the energy metabolism of Ehrlich ascites tumor cells. *Experimental and Molecular Pathology*.

[13] Newell DR, Mansi J, Hardy J (1991). The pharmacokinetics of oral lonidamine in breast and lung cancer patients. *Seminars in Oncology*.

[14] Graziani Y (1977). Bioflavonoid regulation of ATPase and hexokinase activity in Ehrlich ascites cell mitochondria. *Biochimica et Biophysica Acta*.

[15] Mamani-Matsuda M, Rambert J, Malvy D (2004). Quercetin Induces Apoptosis of *Trypanosoma brucei gambiense* and decreases the proinflammatory response of human macrophages. *Antimicrobial Agents and Chemotherapy*.

[16] Dodson HC, Lyda TA, Chambers JW, Morris MT, Christensen KA, Morris JC (2011). Quercetin, a fluorescent bioflavanoid, inhibits *Trypanosoma brucei* hexokinase 1. *Experimental Parasitology*.

[17] Hudock MP, Sanz-Rodríguez CE, Song Y (2006). Inhibition of *Trypanosoma cruzi* hexokinase by bisphosphonates. *Journal of Medicinal Chemistry*.

[18] Sanz-Rodríguez CE, Concepción JL, Pekerar S, Oldfield E, Urbina JA (2007). Bisphosphonates as inhibitors of *Trypanosoma cruzi* hexokinase: kinetic and metabolic studies. *Journal of Biological Chemistry*.

[19] Sharlow ER, Lyda TA, Dodson HC (2010). A target-based high throughput screen yields *Trypanosoma brucei* hexokinase small molecule inhibitors with antiparasitic activity. *PLoS Neglected Tropical Diseases*.

[20] Bakker BM, Michels PAM, Opperdoes FR, Westerhoff HV (1997). Glycolysis in bloodstream form *Trypanosoma brucei* can be understood in terms of the kinetics of the glycolytic enzymes. *Journal of Biological Chemistry*.

[21] Morris MT, DeBruin C, Yang Z, Chambers JW, Smith KS, Morris JC (2006). Activity of a second *Trypanosoma brucei* hexokinase is controlled by an 18-amino-acid C-terminal tail. *Eukaryotic Cell*.

[22] Verlinde CLMJ, Hannaert V, Blonski C (2001). Glycolysis as a target for the design of new anti-trypanosome drugs. *Drug Resistance Updates*.

[23] Hornberg JJ, Bruggeman FJ, Bakker BM, Westerhoff HV (2007). Metabolic control analysis to identify optimal drug targets. *Progress in Drug Research*.

[24] Morris JC, Wang Z, Drew ME, Englund PT (2002). Glycolysis modulates trypanosome glycoprotein expression as revealed by an RNAi library. *EMBO Journal*.

[25] Colasante C, Ellis M, Ruppert T, Voncken F (2006). Comparative proteomics of glycosomes from bloodstream form and procyclic culture form *Trypanosoma brucei brucei*. *Proteomics*.

[26] Chudzik DM, Michels PA, De Walque S, Hol WGJ (2000). Structures of type 2 peroxisomal targeting signals in two trypanosomatid aldolases. *Journal of Molecular Biology*.

[27] Azéma L, Lherbet C, Baudoin C, Blonski C (2006). Cell permeation of a *Trypanosoma brucei* aldolase inhibitor: evaluation of different enzyme-labile phosphate protecting groups. *Bioorganic and Medicinal Chemistry Letters*.

[28] Cáceres AJ, Michels PAM, Hannaert V (2010). Genetic validation of aldolase and glyceraldehyde-3-phosphate dehydrogenase as drug targets in *Trypanosoma brucei*. *Molecular and Biochemical Parasitology*.

[29] Galland N, de Walque S, Voncken FGJ, Verlinde CLMJ, Michels PAM (2010). An internal sequence targets *Trypanosoma brucei* triosephosphate isomerase to glycosomes. *Molecular and Biochemical Parasitology*.

[30] Helfert S, Estévez AM, Bakker B, Michels P, Clayton C (2001). Roles of triosephosphate isomerase and aerobic metabolism in *Trypanosoma brucei*. *Biochemical Journal*.

[31] Kohl L, Drmota T, Do Thi CD (1996). Cloning and characterization of the NAD-linked glycerol-3-phosphate dehydrogenases of *Trypanosoma brucei brucei* and *Leishmania mexicana mexicana* and expression of the trypanosome enzyme in *Escherichia coli*. *Molecular and Biochemical Parasitology*.

[32] Aronov AM, Suresh S, Buckner FS (1999). Structure-based design of submicromolar, biologically active inhibitors of trypanosomatid glyceraldehyde-3-phosphate dehydrogenase. *Proceedings of the National Academy of Sciences of the United States of America*.

[33] Peterson GC, Sommer JM, Klosterman S, Wang CC, Parsons M (1997). *Trpanosoma brucei*: identification of an internal region of phosphoglycerate kinase required for targeting to glycosomal microbodies. *Experimental Parasitology*.

[34] Alexander K, Parail AC, Parsons M (1990). An allele of *Trypanosoma brucei* cytoplasmic phosphoglycerate kinase is a mosaic of other alleles and genes. *Molecular and Biochemical Parasitology*.

[35] Subramaniam C, Veazey P, Redmond S (2006). Chromosome-wide analysis of gene function by RNA interference in the African trypanosome. *Eukaryotic Cell*.

[36] Bressi JC, Choe J, Hough MT (2000). Adenosine analogues as inhibitors of *Trypanosoma brucei* phosphoglycerate kinase: elucidation of a novel binding mode for a 2-Amino-N(6)-substituted adenosine. *Journal of Medicinal Chemistry*.

[37] Furuya T, Kessler P, Jardim A, Schnaufer A, Crudder C, Parsons M (2002). Glucose is toxic to glycosome-deficient trypanosomes. *Proceedings of the National Academy of Sciences of the United States of America*.

[38] Kessler PS, Parsons M (2005). Probing the role of compartmentation of glycolysis in procyclic form *Trypanosoma brucei*: RNA interference studies of PEX14, hexokinase, and phosphofructokinase. *Journal of Biological Chemistry*.

[39] Haanstra JR, Van Tuijl A, Kessler P (2008). Compartmentation prevents a lethal turbo-explosion of glycolysis in trypanosomes. *Proceedings of the National Academy of Sciences of the United States of America*.

[40] Kumar R, Gupta S, Srivastava R, Sahasrabuddhe AA, Gupta CM (2010). Expression of a PTS2-truncated hexokinase produces glucose toxicity in *Leishmania donovani*. *Molecular and Biochemical Parasitology*.

[41] Gould SJ, Keller GA, Hosken N, Wilkinson J, Subramani S (1989). A conserved tripeptide sorts proteins to peroxisomes. *Journal of Cell Biology*.

[42] Gatto GJ, Geisbrecht BV, Gould SJ, Berg JM (2000). Peroxisomal targeting signal-1 recognition by the TPR domains of human PEX5. *Nature Structural Biology*.

[43] Tsukamoto T, Hata S, Yokota S (1994). Characterization of the signal peptide at the amino terminus of the rat peroxisomal 3-ketoacyl-CoA thiolase precursor. *Journal of Biological Chemistry*.

[44] Glover JR, Andrews DW, Subramani S, Rachubinski RA (1994). Mutagenesis of the amino targeting signal of *Saccharomyces cerevisiae* 3- ketoacyl-CoA thiolase reveals conserved amino acids required for import into peroxisomes in vivo. *Journal of Biological Chemistry*.

[45] Small GM, Szabo LJ, Lazarow PB (1988). Acyl-CoA oxidase contains two targeting sequences each of which can mediate protein import into peroxisomes. *EMBO Journal*.

[46] Marzioch M, Erdmann R, Veenhuis M, Kunau WH (1994). PAS7 encodes a novel yeast member of the WD-40 protein family essential for import of 3-oxoacyl-CoA thiolase, a PTS2-containing protein, into peroxisomes. *EMBO Journal*.

[47] Zhang JW, Lazarow PB (1995). PEB1 (PAS7) in *Saccharomyces cerevisiae* encodes a hydrophilic, intra- peroxisomal protein that is a member of the WD repeat family and is essential for the import of thiolase into peroxisomes. *Journal of Cell Biology*.

[48] Rehling P, Marzioch M, Niesen F, Wittke E, Veenhuis M, Kunau WH (1996). The import receptor for the peroxisomal targeting signal 2 (PTS2) in *Saccharomyces cerevisiae* is encoded by the PAS7 gene. *EMBO Journal*.

[49] Braverman N, Dodt G, Gould SJ, Valle D (1998). An isoform of Pex5p, the human PTS1 receptor, is required for the import of PTS2 proteins into peroxisomes. *Human Molecular Genetics*.

[50] Otera H, Okumoto K, Tateishi K (1998). Peroxisome targeting signal type 1 (PTS1) receptor is involved in import of both PTS1 and PTS2: studies with PEX5-defective CHO cell mutants. *Molecular and Cellular Biology*.

[51] Pilar AVC, Madrid KP, Jardim A (2008). Interaction of Leishmania PTS2 receptor peroxin 7 with the glycosomal protein import machinery. *Molecular and Biochemical Parasitology*.

[52] Galland N, Demeure F, Hannaert V (2007). Characterization of the role of the receptors PEX5 and PEX7 in the import of proteins into glycosomes of *Trypanosoma brucei*. *Biochimica et Biophysica Acta*.

[53] Sampathkumar P, Roach C, Michels PAM, Hol WGJ (2008). Structural insights into the recognition of peroxisomal targeting signal 1 by *Trypanosoma brucei* peroxin 5. *Journal of Molecular Biology*.

[54] Parsons M, Furuya T, Pal S, Kessler P (2001). Biogenesis and function of peroxisomes and glycosomes. *Molecular and Biochemical Parasitology*.

[55] Opperdoes FR (1987). Compartmentation of carbohydrate metabolism in trypanosomes. *Annual Review of Microbiology*.

[56] Hoepfner D, Schildknegt D, Braakman I, Philippsen P, Tabak HF (2005). Contribution of the endoplasmic reticulum to peroxisome formation. *Cell*.

[57] Van Der Zand A, Braakman I, Tabak HF (2010). Peroxisomal membrane proteins insert into the endoplasmic reticulum. *Molecular Biology of the Cell*.

[58] South ST, Gould SJ (1999). Peroxisome synthesis in the absence of preexisting peroxisomes. *Journal of Cell Biology*.

[59] Kim PK, Mullen RT, Schumann U, Lippincott-Schwartz J (2006). The origin and maintenance of mammalian peroxisomes involves a de novo PEX16-dependent pathway from the ER. *Journal of Cell Biology*.

[60] Herman M, Gillies S, Michels PA, Rigden DJ (2006). Autophagy and related processes in trypanosomatids: insights from genomic and bioinformatic analyses. *Autophagy*.

[61] Banerjee SK, Kessler PS, Saveria T, Parsons M (2005). Identification of trypanosomatid PEX19: functional characterization reveals impact on cell growth and glycosome size and number. *Molecular and Biochemical Parasitology*.

[62] Saveria T, Halbach A, Erdmann R (2007). Conservation of PEX19-binding motifs required for protein targeting to mammalian peroxisomal and trypanosome glycosomal membranes. *Eukaryotic Cell*.

[63] Erdmann R, Blobel G (1995). Giant peroxisomes in oleic acid-induced *Saccharomyces cerevisiae* lacking the peroxisomal membrane protein Pmp27p. *Journal of Cell Biology*.

[64] Marshall PA, Krimkevich YI, Lark RH, Dyer JM, Veenhuis M, Goodman JM (1995). Pmp27 promotes peroxisomal proliferation. *Journal of Cell Biology*.

[65] Rottensteiner H, Stein K, Sonnenhol E, Erdmann R (2003). Conserved function of Pex11p and the novel Pex25p and Pex27p in peroxisome biogenesis. *Molecular Biology of the Cell*.

[66] Orth T, Reumann S, Zhang X (2007). The PEROXIN11 protein family controls peroxisome proliferation in Arabidopsis. *Plant Cell*.

[67] Abe I, Fujiki Y (1998). cDNA cloning and characterization of a constitutively expressed isoform of the human peroxin Pex11p. *Biochemical and Biophysical Research Communications*.

[68] Li X, Gould SJ (2002). PEX11 promotes peroxisome division independently of peroxisome metabolism. *Journal of Cell Biology*.

[69] Lorenz P, Maier AG, Baumgart E, Erdmann R, Clayton C (1998). Elongation and clustering of glycosomes in *Trypanosoma brucei* overexpressing the glycosomal Pex11p. *EMBO Journal*.

[70] Maier A, Lorenz P, Voncken F, Clayton C (2001). An essential dimeric membrane protein of trypanosome glycosomes. *Molecular Microbiology*.

[71] Marshall PA, Dyer JM, Quick ME, Goodman JM (1996). Redox-sensitive homodimerization of Pex11p: a proposed mechanism to regulate peroxisomal division. *Journal of Cell Biology*.

[72] Knoblach B, Rachubinski RA (2010). Phosphorylation-dependent activation of peroxisome proliferator protein PEX11 controls peroxisome abundance. *Journal of Biological Chemistry*.

[73] Passreiter M, Anton M, Lay D (1998). Peroxisome biogenesis: involvement of ARF and coatomer. *Journal of Cell Biology*.

[74] Saleem RA, Knoblach B, Mast FD (2008). Genome-wide analysis of signaling networks regulating fatty acid-induced gene expression and organelle biogenesis. *Journal of Cell Biology*.

[75] Saraya R, Veenhuis M, Van Der Klei IJ (2010). Peroxisomes as dynamic organelles: peroxisome abundance in yeast. *FEBS Journal*.

[76] Nagotu S, Veenhuis M, Van der Klei IJ (2010). Divide et impera: the dictum of peroxisomes. *Traffic*.

[77] Kuravi K, Nagotu S, Krikken AM (2006). Dynamin-related proteins Vps1p and Dnm1p control peroxisome abundance in *Saccharomyces cerevisiae*. *Journal of Cell Science*.

[78] Morgan GW, Goulding D, Field MC (2004). The single dynamin-like protein of *Trypanosoma brucei* regulates mitochondrial division and is not required for endocytosis. *Journal of Biological Chemistry*.

[79] Chanez AL, Hehl AB, Engstler M, Schneider A (2006). Ablation of the single dynamin of *T. brucei* blocks mitochondrial fission and endocytosis and leads to a precise cytokinesis arrest. *Journal of Cell Science*.

[80] Koch A, Yoon Y, Bonekamp NA, McNiven MA, Schrader M (2005). A role for Fis1 in both mitochondrial and peroxisomal fission in mammalian cells. *Molecular Biology of the Cell*.

[81] Zhang X, Hu J (2009). Two small protein families, DYNAMIN-RELATED PROTEIN3 and FISSION1, are required for peroxisome fission in Arabidopsis. *Plant Journal*.

[82] Motley AM, Ward GP, Hettema EH (2008). Dnm1p-dependent peroxisome fission requires Caf4p, Mdv1p and Fis1p. *Journal of Cell Science*.

[83] Kobayashi S, Tanaka A, Fujiki Y (2007). Fis1, DLP1, and Pex11p coordinately regulate peroxisome morphogenesis. *Experimental Cell Research*.

[84] Vizeacoumar FJ, Vreden WN, Fagarasanu M, Eitzen GA, Aitchison JD, Rachubinski RA (2006). The dynamin-like protein Vps1p of the yeast *Saccharomyces cerevisiae* associates with peroxisomes in a Pex19p-dependent manner. *Journal of Biological Chemistry*.

[85] Herman M, Pérez-Morga D, Schtickzelle N, Michels PAM (2008). Turnover of glycosomes during life-cycle differentiation of *Trypanosoma brucei*. *Autophagy*.

